# Leukocyte telomere length, T cell composition and DNA methylation age

**DOI:** 10.18632/aging.101293

**Published:** 2017-09-20

**Authors:** Brian H. Chen, Cara L. Carty, Masayuki Kimura, Jeremy D. Kark, Wei Chen, Shengxu Li, Tao Zhang, Charles Kooperberg, Daniel Levy, Themistocles Assimes, Devin Absher, Steve Horvath, Alexander P. Reiner, Abraham Aviv

**Affiliations:** ^1^ Longitudinal Studies Section, Translational Gerontology Branch, Intramural Research Program, National Institute on Aging, National Institutes of Health, Baltimore, MD 21224, USA; ^2^ Framingham Heart Study, National Heart, Lung, and Blood Institute, Framingham, MA 01702, USA; ^3^ Population Sciences Branch, Division of Intramural Research, National Heart, Lung and Blood Institute, Bethesda, MD 20892, USA; ^4^ Division of Biostatistics and Study Methodology, Center for Translational Science, George Washington University and Children's National Medical Center, Washington, DC 20010, USA; ^5^ Center of Development and Aging, New Jersey Medical School, Rutgers State University of New Jersey, Newark, NJ 07103, USA; ^6^ Epidemiology Unit, Hebrew University-Hadassah School of Public Health and Community Medicine, Jerusalem, Israel; ^7^ Department of Epidemiology, School of Public Health and Tropical Medicine, Tulane University, New Orleans, LA 70118, USA; ^8^ Division of Public Health Sciences, Fred Hutchinson Cancer Research Center, Seattle, WA 98109, USA; ^9^ Department of Medicine, Stanford University School of Medicine, Stanford, CA 94305, USA; ^10^ HudsonAlpha Institute for Biotechnology, Huntsville, AL 35806, USA; ^11^ Human Genetics, David Geffen School of Medicine, University of California Los Angeles, Los Angeles, CA 90095, USA; ^12^ Biostatistics, School of Public Health, University of California Los Angeles, Los Angeles, CA 90095, USA; ^13^ Department of Epidemiology, University of Washington, Seattle, WA 98195, USA

**Keywords:** telomeres, aging, T cells, DNA methylation, memory, naïve

## Abstract

Both leukocyte telomere length (LTL) and DNA methylation age are strongly associated with chronological age. One measure of DNA methylation age-the extrinsic epigenetic age acceleration (EEAA)-is highly predictive of all-cause mortality. We examined the relation between LTL and EEAA. LTL was measured by Southern blots and leukocyte DNA methylation was determined using Illumina Infinium HumanMethylation450 BeadChip in participants in the Women's Health Initiative (WHI; n=804), the Framingham Heart Study (FHS; n=909) and the Bogalusa Heart study (BHS; n=826). EEAA was computed using 71 DNA methylation sites, further weighted by proportions of naïve CD8^+^ T cells, memory CD8^+^ T cells, and plasmablasts. Shorter LTL was associated with increased EEAA in participants from the WHI (*r*=-0.16, *p*=3.1x10^−6^). This finding was replicated in the FHS (*r*=-0.09, *p*=6.5x10^−3^) and the BHS (*r*=−0.07, *p*=3.8x 10^−2^). LTL was also inversely related to proportions of memory CD8^+^ T cells (*p*=4.04x10^−16^) and positively related to proportions of naive CD8^+^ T cells (*p*=3.57x10^−14^). These findings suggest that for a given age, an individual whose blood contains comparatively more memory CD8^+^ T cells and less naive CD8^+^ T cells would display a relatively shorter LTL and an older DNA methylation age, which jointly explain the striking ability of EEAA to predict mortality.

## INTRODUCTION

Aging eludes precise definition at the systemic level and denotes a multitude of processes at the cellular level. Two of these processes-age-dependent telomere shortening [1] and DNA methylation (DNAm) profiles of cytosine phosphate guanines (CpGs) [2-4] have been used as indices of biological age. The age estimates resulting from multivariable regression models of DNAm profiles are referred to as ‘DNAm age’ or ‘epigenetic age’.

The discrepancy between DNAm age and chronological age is an estimate of the ‘epigenetic age acceleration’, which has been found to increase in Down syndrome [5], obesity [6], HIV [7] and early menopause [8]. Notably, measures of epigenetic age in blood have been reported to be predictive of all-cause mortality after adjusting for chronological age and traditional risk factors such as sex, hypertension, and prior history of disease [9-11]. A recent meta-analysis showed that among several estimates of epigenetic age acceleration, one particular measure, i.e., extrinsic epigenetic age acceleration (EEAA), was superior in predicting all-cause mortality [10], but the reason for this has remained unclear. EEAA is defined as the weighted average of DNAm age and imputed proportions of naïve CD8^+^ T cells, memory CD8^+^ T cells and plasmablasts [12]. Here we show a novel correlation between leukocyte telomere length (LTL) and EEAA. We infer that this correlation reflects the aging of the immune system, as expressed in the age-dependent change of the proportions of naive CD8^+^ T cells and memory CD8^+^ T cells.

## RESULTS

Major characteristics of participants from the WHI (the discovery cohort), the FHS and the BHS are displayed in Table S1 and Figure S1 (available as Supplementary data on line).

**Figure 1:**
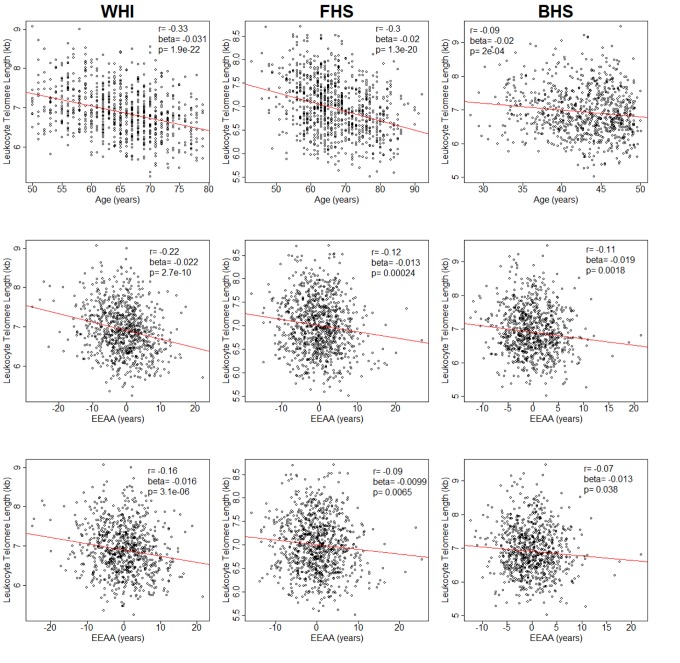
Plots of leukocyte telomere length (LTL) against chronological age (upper row) and extrinsic epigenetic age acceleration (EEAA) (second and third rows) Second row displays unadjusted EEAA. Third row displays EEAA adjusted for BMI, sex, race/ethnicity, and current smoking status. First column displays associations for the Women's Health Initiative (WHI, n=804). Second column displays associations for the Framingham Heart Study (FHS, n=909). Third column displays associations for the Bogalusa Heart Study (BHS, n=826).

In WHI, LTL was negatively correlated with chronological age (*r*= −0.33, *p* = 1.9 x 10^−22^) (Figure 1). LTL, adjusted for age, was also negatively correlated with EEAA (*r* = −0.22, *p* = 2.7 x 10^−10^). This correlation persisted after further adjustment for race/ethnicity, sex, BMI and current smoking status (*r* = −0.16, *p* = 3.1 x 10^−6^) and was replicated in both FHS (*r* = −0.09, *p* = 6.5 x 10^−3^) and BHS (*r* = −0.07, *p* = 3.8 x 10^−2^) (Figure 1). In sensitivity analyses using the WHI sample, the relationship remained significant (*p* = 0.005) after additional adjustment for the covariates: systolic and diastolic blood pressure, education level, income, diabetes, high density lipoprotein cholesterol, low density lipoprotein cholesterol, triglycerides, and C-reactive protein. Tests for interaction showed no differences in LTL and EEAA associations by sex (Table S2) or race/ethnicity (Table S3) after adjusting for age, BMI, and current smoking status. Thus, subsequent analyses were conducted on the pooled data from all three cohorts.

As EEAA was built on specific 71 CpG sites described by Hannum et al. [3] and further modified for imputed proportions of naïve CD8^+^ T cells, memory CD8^+^ T cells, and plasmablasts [12], its correlation with LTL may be due to an intrinsic property of the CpG sites, the leukocyte proportions, or both.

We therefore examined (first in WHI and then in FHS and BHS) the relationship between LTL and imputed proportions of these three cell populations. In WHI, the proportion of naïve CD8^+^ T cells was positively correlated with LTL (*r* = 0.19, *p* = 2.84 x 10^−8^) after adjusting for age, sex, BMI, race/ethnicity, and current smoking status (Table 1). This finding was consistent in the two replication cohorts (*r* = 0.19, *p* = 3.53 x 10^−9^ in FHS and *r* = 0.21, *p* = 2.47 x 10^−9^ in BHS). The proportion of memory CD8^+^ T cells was negatively correlated with LTL in WHI (*r* = −0.20, *p* =1.76 x 10^−8^) and the replication cohorts (*r* = −0.16, *p* =1.06 x 10^−6^ in FHS, and *r* = −0.18, *p* =1.50 x 10^−7^ in BHS). Plasmablast proportion was negatively correlated with LTL in WHI (*r* = −0.09, *p* = 0.01) but was not significant in the replication cohorts (*r* = 0.03, *p* = 0.41 in FHS and *r* = 0.03, *p* = 0.35 in BHS). No sex or racial/ethnic differences were detected in any of these correlations (*p* > 0.05).

**Table 1:** Partial correlation coefficients (Pearson) and linear regression coefficients for associations of leukocyte telomere length with blood cell subpopulations in three cohorts (WHI, FHS, BHS)

		CD8^+^ naïve	CD8^+^ memory	Plasmablasts
WHI*	*r* (Pearson)	0.19	−0.20	−0.09
	beta	0.0023	−0.031	−0.229
	*p*-value	2.8x10^−8^	1.8x10^−8^	0.01
FHS	*r* (Pearson)	0.19	−0.16	0.03
	beta	0.0026	−0.025	0.076
	*p*-value	3.5x10^−9^	1.1x10^−6^	0.41
BHS	*r* (Pearson)	0.21	−0.18	0.03
	beta	0.0037	−0.038	0.148
	*p*-value	2.5x10^−9^	1.5x10^−7^	0.35
Meta-analysis^†^	*r* (Pearson)	0.20	−0.18	−0.01
	beta	0.0027	−0.030	−0.018
	*p*-value	3.6x10^−14^	4.0 x10^−16^	0.88

* Discovery cohort; ^†^Meta-analysis of correlation coefficients was conducted using the DerSimonian-Laird random-effects meta-analytical approach. Meta-analysis of linear regression beta coefficients was conducted using a random effects model with the DerSimonian-Laird estimator. WHI = Women's Health Initiative; FHS = Framingham Heart Study; BHS = Bogalusa Heart Study. All associations adjusted for age, sex, BMI, race/ethnicity, and current smoking status (regression model: Telomere length in kb = Cell Proportion + age (in FHS and BHS) + sex + BMI + race (in WHI and BHS) + current smoking).

Meta-analyses, combining the three cohorts (Table 1), showed that after adjustment for age, sex, race/ethnicity, BMI and current smoking status, LTL was negatively correlated, r = −0.12, with the EEAA at p = 7.32 x 10^−5^, positively correlated with the proportion of naïve CD8^+^ T cells, r = 0.20, p = 3.57 x 10^−14^, and negatively correlated with the proportion of memory CD8^+^ cells, r = −0.18, p = 4.04 x 10^−16^. No significant correlations were found between LTL and the proportion of plasmablasts.

Chen et al. introduced a measure of epigenetic age acceleration that was independent of cell proportions, known as IEAA [10]. We examined the relation between LTL and two versions of IEAA—one using the Horvath set of CpGs [13] and one using the Hannum et al. CpGs [3]. The IEAA using the Hannum CpGs was not associated with LTL (WHI: *r* = −0.05, *p* = 0.16; FHS: *r* = 0.01, *p* = 0.88; BHS: *r* = 0.02, *p* = 0.66). The IEAA using the Horvath CpGs was not associated with LTL in WHI (*r* = −0.05, *p* = 0.12) and FHS (*r* = 0, *p* = 0.95) but was significant in BHS (*r* = 0.08, *p* = 0.016).

Finally, we performed two additional sets of analyses to ascertain that the correlation between LTL and EAAA arises from correlation between LTL and CD8 ^+^ T cells. First, we adjusted for the proportions naïve CD8^+^ T cells, memory CD8^+^ cells and plasmablasts. This led to non-significant correlations between LTL and EEAA in all cohorts (WHI: r = −0.04, p = 0.28; FHS: r = 0, p = 0.99; BHS: r = 0.04, p = 0.31). Second, we also examined EEAA using another set of CpG sites, described by Horvath [14]. This latter measure of EEAA showed similar associations in WHI (r = −0.18, p = 1.9 x 10-^7^) and FHS (r = −0.11, p = 1.1 x 10-^3^) but was not significant in BHS (r = −0.03, p = 0.36).

## DISCUSSION

The two key observations of this study are: (a) LTL is inversely correlated with EEAA; and (b) the LTL-EEAA correlation largely reflects the proportions of imputed naïve and memory CD8^+^ T cell populations in the leukocytes from which DNA was extracted. These correlations were independently replicated in two well-characterized cohorts, providing confidence in their validity. To our knowledge, this is the first study showing association between LTL and a specific formulation of the epigenetic age, but only when it was weighted by the proportions of T naïve cells, T memory cells and plasmoblats (i.e., the EEAA). A previous study, using the Hannum formulation [3], showed no significant association between LTL and epigenetic age [14]. Overall, these findings might explain the ability of EEAA to predict all-cause mortality, given that EEAA captures not only leukocyte DNAm age but also a key aspect of immune senescence (principally naïve and memory T cells), which increases risks of a host of age-related diseases and of death [15].

TL in every leukocyte lineage generally reflects the individual's TL across somatic cells [16,17], which is highly heritable [18,19], but highly variable between individuals. Such variability (SD ~ 0.7 kb) is already displayed across newborns [20-22]. After birth, TL shortening in leukocyte lineages reflects hematopoietic stem cell replication. In a subset of leukocyte lineages, replication continues in sites outside the bone marrow, including the thymus and secondary lymphoid organs, where antigenic stimulation induces their further proliferation and differentiation [23].

The involution of the thymus with aging brings about the progressive, age-dependent decline in the proportion of naïve CD8^+^ T cells with the concomitant increase in the proportion of memory CD8^+^ T cells (Figure S2) [23-26]. Decreasing naïve T-cell number may affect immune function and competence and in part explains the declining cellular immune function observed with aging [27]. For example, both the number and diversity of T cell populations correlate with vaccine response and resistance to opportunistic infections [28,29]. As TL is shorter and (*in vitro*) proliferative potential is compromised in memory compared to naïve cells [21,30], LTL would be comparatively shorter when a high proportion of memory CD8^+^ T cells is present in a sample of leukocytes. In contrast, LTL would be comparatively longer when a high proportion of naive CD8^+^ T cells is present in the sample. From this perspective, for a given age, an individual with com-paratively more memory CD8^+^ T cells and less naive CD8^+^ T cells appears to have an older biological profile of the immune system; such an individual also displays a shorter age-adjusted LTL and an older EEAA profile.

This inference has considerable ramifications for the two competing views about the biological meaning of LTL dynamics (LTL at birth and its shortening thereafter).

The first and more popular view considers LTL as a biomarker— a ‘telomeric clock’— of human aging. However, given that LTL variation across newborns is as wide as that in adults [20-22], the ‘telomeric clock’ does not start at the same zero ‘biological time’ in different individuals [31].

For this reason, the second view suggests that although in itself LTL is an inadequate marker of human aging, it can forecast major aging-related diseases [32]. As LTL is highly heritable [18,19], having constitutively short (or long) telomeres precedes the onset of LTL-associated diseases by decades [33,34]. It is thus likely that TL might play an active role in disease development. This conjecture is supported by findings that not only LTL but also LTL-associated alleles are associated with the incidence of two major disease categories— cardiovascular disease and cancer [32,35]. Such findings largely exclude reverse causality, i.e., the possibility that cardiovascular disease, major cancers or their underlying causes bring about changes in LTL.

These competing interpretations of the biological meaning of LTL are not mutually exclusive for the following reasons: Because of wide LTL variation between newborns, only a fraction of the inter-individual variation in LTL between adults reflects variation in age-dependent LTL shortening after birth. Herein lies the relevance of the correlations of LTL with EEAA and with the relative numbers of naïve and memory CD8^+^ T cells. As TL shortening in T cells of adults reflects their antigen-mediated replicative histories, the associations of LTL with EEAA (and naïve and memory CD8^+^ T cells) suggest that the shortening of LTL with age captures, in part, the aging of the immune system. Thus, age-dependent variation in LTL shortening might partially record different histories of the immune responses in different individuals under different environmental settings.

Notably, in the FHS, LTL and DNAm were measured 10 years apart (Materials and Methods), but we doubt that this 10-year gap had major influence on the findings, given that LTL in adults displays strong tracking, such that individuals maintain their comparative LTL ranking throughout adulthood [33,34]. In fact, the age-dependent trajectories of not only LTL [17,33,34] but also DNAm age [36] are largely determined prior to adulthood. As the immune system is primarily fashioned during early life [37], it is reasonable to propose that to gain further mechanistic insight, the focus of studying both LTL and epigenetic age should be shifted from adults to children.

Finally, our findings are based on imputation using the DNA Methylation Age Calculator (Materials and Methods) rather than direct measurements of the numbers of T cells. There is no reason to believe that direct measurements of T cells would have generated different conclusions, albeit the absolute values of the LTL-EEAA might have been slightly different. In fact, DNAm profiling may provide a valuable tool to follow LTL dynamics under different environmental settings in relation to changing proportions of naïve and memory CD8^+^ T cells without the necessity to resort to direct measurements of the numbers of these cells. Such an approach may enable stored DNA samples to be reexamined for the study of LTL and lymphocyte population dynamics.

## MATERIALS AND METHODS

Participants originated from the Women's Health Initiative (WHI), the Framingham Heart Study (FHS) and the Bogalusa Heart Study (BHS); all signed informed consents approved by respective institutional review boards. All participants consented for the use of their DNA in genetic research. Analytic codes can be obtained from authors (BHC, CLC) upon request.

### Women's Health Initiative

Details on the WHI have been published previously [38-40]. The cohort comprised white (of European ancestry) and African American postmenopausal women with both LTL and DNAm age measures in blood samples collected at baseline (1993-98). These women were part of two WHI ancillary studies measuring LTL or DNAm age. Data are available from this page: https://www.whi.org/researchers/Stories/June%202015%20WHI%20Investigators'%20Datasets%20Released.aspx; also see the following link: https://www.whi.org/researchers/data/Documents/WHI%20Data%20Preparation%20and%20Use.pdf

### Framingham Heart Study

The FHS Offspring Cohort began enrollment in 1971 and included offspring and spouses of the offspring of the FHS original cohort. LTL was measured in samples from the sixth examination (1995-1998); DNAm analysis was performed on samples from the eighth examination (2005-2008). These populations were described previously [41-43]. The FHS data are available in dbGaP (accession number "phs000724.v2.p9").

### Bogalusa Heart Study

The BHS is a study of the natural history of cardiovascular disease beginning in childhood in the biracial community (65% white, 35% African American) of Bogalusa, Louisiana [44]. LTL data were available for participants, who had blood samples collected on 2 occasions, a baseline examination in 1995–1996 and a follow-up examination in 2001–2006. The LTL and DNAm analyses were performed on samples from the latter examination. The longitudinal cardiovascular risk factor phenotype and genotype data of the BHS cohort are available via application through the NHLBI Biologic Specimen and Data Repository Information Coordinating Center website (https://biolincc.nhlbi.nih.gov/studies/bhs). The longitu-dinal datasets of risk factor variables since childhood, calculated variables, LTL and genome-wide DNA methylation data generated from the proposed study will be made available to outside researchers on this website.

### Leukocyte telomere length measurements

LTL was measured by the mean length terminal restriction fragments using the Southern blot method, as previously described [45]. The inter-assay coefficient of variation for blinded pair sets was 2.0% for the WHI, 1.4% for the BHS and 2.4% for the FHS.

### Extrinsic Epigenetic Age Acceleration (EEAA) and Intrinsic Epigenetic Age Acceleration (IEAA)

EEAA was defined as the residual variation resulting from a univariate model regressing the epigenetic age described by Hannum et al. on chronological age [3], which was further weighted by the proportions of 3 cell types: naïve (CD8^+^CD45RA^+^CCR7^+^) T cells, memory (CD8^+^CD28^−^CD45RA^−^) T cells, and plasmablasts; the weights were determined by the correlation between the respective variable and chronological age [46]. The cell proportions were estimated from the DNAm data, as implemented in the online DNA Methylation Age Calculator (https://dnamage.genetics.ucla.edu/).

By construction, EEAA is positively correlated with the memory CD8^+^ T cells, plasmablast cells, and negatively correlated with naïve CD8^+^ T cells. Thus, EEAA captures both age-related DNAm changes and age-related changes in the composition of naïve T cells, memory T cells and plasmablasts.

We have also examined the correlation between LTL and IEAA, which was calculated as the residual resulting from multivariate regression of the epigenetic age on chronological age and estimated numbers of naive CD8^+^ T cells, memory CD8^+^ T cells, plasma-blasts, CD4^+^ T cells, natural killer cells, monocytes, and granulocytes. By definition, IEAA is not correlated with chronological age and is, at most, only very weakly correlated with measures of leukocyte counts. IEAA is meant to capture properties of the aging process that exhibit some preservation across various cell and tissue types and organs.

### DNA methylation quantification

Bisulfite treated genomic DNA was hybridized to the Illumina Infinium HumanMethylation450 BeadChip (Illumina, Inc, San Diego, CA, USA). Background-corrected DNAm beta values were uploaded to the online DNA Methylation Age Calculator to obtain age acceleration measures.

### Statistical analysis

Cohort differences for continuous and categorical variables were tested using one-way ANOVA or Fisher's exact tests, respectively. Sex and race interactions were tested using a Wald test. Beta coefficients were estimated using linear regression with DNAm age as the dependent variable and LTL as the independent variable, adjusted for age, body mass index (BMI), and current smoking status. Partial correlation coefficients were adjusted for age, BMI, and current smoking status using the pcor command in the ppcor R package [47]. Meta-analysis of correlation coefficients was conducted using the DerSimonian-Laird random-effects meta-analytical approach using the metacor.DSL command in the metacor R package. Meta-analysis of linear regression estimates was conducted using the DerSimonian-Laird random-effects model implemented in the rma command in the metafor R package. Tests for differences between two correlations was con-ducted using the r. test command in the psych R package [48].

## SUPPLEMENTARY MATERIAL TABLES AND FIGURES


