# Supplementary material for: Leukocyte telomere length, T cell composition and DNA methylation age
Source: Aging (Albany NY). 2017 Sep 20;9(9):1983–95. doi: 10.18632/aging.101293 (PMC5636670; doi:10.18632/aging.101293)
Supplement: Supplementary file 1 [file aging-09-1983-s001.pdf]

SUPPLEMENTAL MATERIAL

Table S1. Study population characteristics.

Variable	WHI	FHS	BHS
Sample size, n	804	909	826
Median age in years, (25th and 75th percentiles)	66 (60, 70)	66 (61, 74)[a]	44 (40, 47)
Female sex (n, %)	804 (100%)	469 (52%)	470 (57%)
Median BMI in kg/m^2 (25th and 75th percentiles)	29.31 (25.51, 33.50)	27.58 (24.69, 30.66)	29.34 (25.62, 34.65)
Current smoker (n, %)	95 (12%)	91 (10%)	224 (27%)
Race/ethnicity (n, %)			
White	467 (58%)	917 (100%)	576 (70%)
African American	337 (42%)	0 (0%)	250 (30%)
Median biomarker distributions (25th and 75th percentiles)			
LTL (kb)	6.87 (6.47, 7.31)	6.96 (6.58, 7.39)	6.85 (6.45, 7.39)
EEAA (years)[†]	-0.83 (-4.94, 2.89)	-0.21 (-3.69, 3.14)	-0.27 (-2.81, 2.72)

[†] Extrinsic epigenetic age acceleration (EEAA) [a] Represents age at sample collection for DNA methylation. Age at LTL sample collection was median=57, 25th percentile=52, and 75th percentile=65 years of age.

Table S2. Sex-specific associations for leukocyte telomere length (kb) and extrinsic epigenetic age acceleration (*years*). All models adjusted for age, body mass index, race (BHS only) and smoking status.

Cohort	Sex	Partial correlation coefficient (p-value)	Beta* (p-value)
FHS	Male	-0.08 (0.09)	-0.78 (0.09)
	Female	-0.11 (0.02)	-1.03 (0.02)
BHS	Male	-0.04 (0.47)	-0.24 (0.47)
	Female	-0.11 (0.02)	-0.63 (0.02)

* Regression model (stratified by sex): EEAA = leukocyte telomere length + age + BMI + race (in BHS) + smoking.**Wald test for differences in beta coefficients across sexes were p=0.58 for FHS and p=0.34 for BHS.

Table S3. Race-specific associations for leukocyte telomere length (kb) and extrinsic epigenetic age acceleration (*years*). All models adjusted for age, body mass index, sex (BHS only) and smoking status.

Cohort	Sex	Partial correlation coefficient (p-value)	Beta* (p-value)
WHI	White	-0.17 (0.0003)	-1.72 (0.0003)
	African American	-0.19 (0.0004)	-2.21 (0.0004)
BHS	White	-0.06 (0.15)	-0.35 (0.15)
	African American	-0.10 (0.12)	-0.65 (0.12)

* Regression model (stratified by race/ethnicity): EEAA = leukocyte telomere length + age + BMI + sex (BHS only) smoking. ** Wald test for differences in beta coefficients across racial/ethnic groups were p= 0.33 for WHI and p=0.50 for BHS.

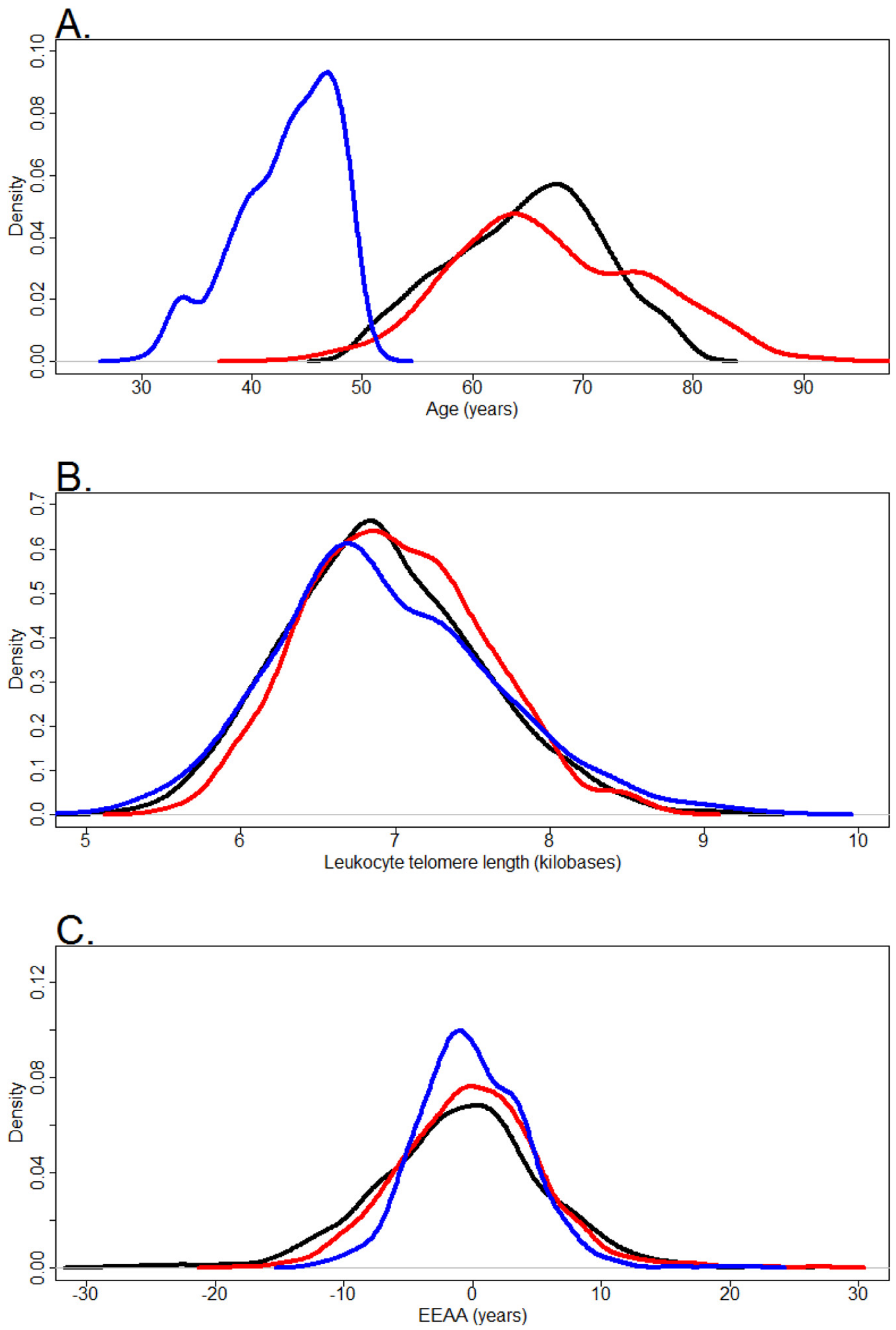

Figure S1. Kernel density distributions across study populations for (A) chronological age, (B) leukocyte telomere length, (C) extrinsic epigenetic age acceleration. Blue=Bogalusa Heart Study. Red=Framingham Heart Study. Black=Women's Health Initiative.

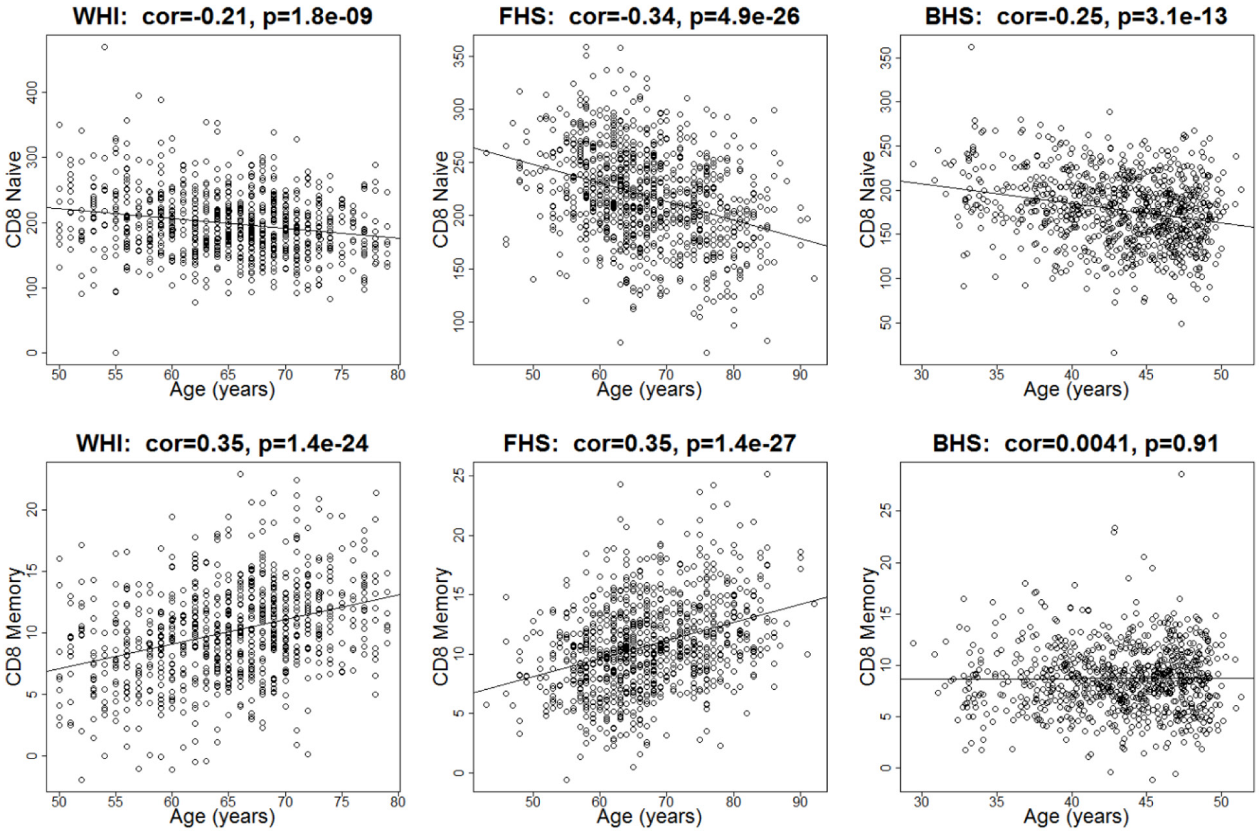

Figure S2. Plots of cell abundances for naïve CD8^{+} T cells (row 1) and memory CD8^{+} T cells (row 2) against chronological age by cohort. Women's Health Initiative (WHI) in column 1. Framingham Heart Study (FHS) in column 2. Bogalusa Heart Study (BHS) in column 3. Cell proportions were estimated from DNA methylation data. The lack of significant correlation in the BHS between memory CD8^{+}T cells and age may be attributable to the younger age and narrower age range in BHS compared to WHI and FHS.
